# The AP2/ERF Transcription Factor ERF56 Negatively Regulating Nitrate-Dependent Plant Growth in *Arabidopsis*

**DOI:** 10.3390/ijms26020613

**Published:** 2025-01-13

**Authors:** Guoqi Yao, Chunhua Mu, Zhenwei Yan, Shijun Ma, Xia Liu, Yue Sun, Jing Hou, Qiantong Liu, Bing Cao, Juan Shan, Bingying Leng

**Affiliations:** 1 Maize Research Institute, Shandong Academy of Agricultural Sciences, Jinan 250100, China; yaoguoqi@saas.ac.cn (G.Y.); maizesd@163.com (C.M.); yanzhenwei@saas.ac.cn (Z.Y.); mashijun@saas.ac.cn (S.M.); liuxia@saas.ac.cn (X.L.); caobing1977@163.com (B.C.); shanjuan@saas.ac.cn (J.S.); 2 College of Agronomy, Qingdao Agricultural University, Qingdao 266109, China; sunyue3070601@163.com (Y.S.); 13356748152@163.com (Q.L.); 3 School of Agriculture, Ludong University, Yantai 264001, China; houjing@m.ldu

**Keywords:** *ERF56*, nitrate, *NLP7*

## Abstract

ERF56, a member of the APETALA2/ETHYLENE-RESPONSIVE FACTOR (AP2/ERF) transcription factor (TF) family, was reported to be an early nitrate-responsive TF in *Arabidopsis*. But the function of *ERF56* in nitrate signaling remains not entirely clear. This study aimed to investigate the role of *ERF56* in nitrate-dependent plant growth and nitrate signaling. We confirmed with reverse transcription quantitative PCR (RT-qPCR) that the transcription of *ERF56* is quickly induced by nitrate. *ERF56* overexpressors displayed decreased nitrate-dependent plant growth, while *erf56* mutants exhibited increased plant growth. Confocal imaging demonstrated that ERF56 is localized into nuclei. Assays with the glucuronidase (GUS) reporter showed that *ERF56* is mainly expressed at the region of maturation of roots and in anthers. The dual-luciferase assay manifested that the transcription of *ERF56* is not directly regulated by NIN-LIKE PROTEIN 7 (NLP7). The transcriptome analysis identified 1038 candidate genes regulated by ERF56 directly. A gene ontology (GO) over-representation analysis showed that *ERF56* is involved in the processes of water transport, inorganic molecule transmembrane transport, secondary metabolite biosynthesis, and cell wall organization. We revealed that *ERF56* represses nitrate-dependent growth through regulating the processes of inorganic molecule transmembrane transport, the secondary metabolite biosynthesis, and cell wall organization.

## 1. Introduction

Nitrogen is one of the three macronutrients for plants. The application of nitrogen fertilizer is one of the most important techniques for modern agriculture production due to the limitation of available nitrogen in the soil. But the production and application of nitrogen fertilizers not only costs a high amount of fossil fuels, but also causes air pollution and eutrophication of aquatic ecosystems [1]. Thus, improving the nitrogen use efficiency (NUE) of crops is urgent for plant breeding.

Nitrate is a predominant nitrogen available for plants in aerated soil [2]. To cope with the high fluctuating nitrate concentration in the soil, plants have evolved a set of mechanisms to regulate nitrate uptake, transport, and metabolism, in which nitrate signaling is involved. Understanding these mechanisms is the base for improving the NUE of crops. Recent years saw a lot of progress in nitrate signaling in plants, especially in *Arabidopsis*. NITRATE TRANSPORTER 1.1 (NRT1.1) was first identified as a nitrate transporter functioning in nitrate uptake [3], and later studies showed NRT1.1 also acts as a sensor to perceive changes in external nitrate and produce a Ca^2+^ signal that is decoded by subgroup III Ca^2+^-SENSOR PROTEIN KINASES (CPKs) [4,5]. After being activated by Ca^2+^, CPKs phosphorylate NIN-LIKE PROTEINS (NLPs) and promote their nuclear localization to specify the reprogramming of genes of downstream pathways [6]. NLPs are master regulators in nitrate signaling in *Arabidopsis* [7,8,9]. Nine NLPs were encoded in the *Arabidopsis* genome, among which NLP7 received the most attention. NLP7 regulates the expression of many nitrate-responsive genes [8], and *nlp7* mutants constitutively show some features of nitrogen-starved plants and are impaired in nitrate signaling [10], indicating that NLP7 is a critical member of NLPs. NLP7 also functions as a nitrate sensor [11]. Many TFs besides NLPs involved in nitrate signaling have been identified [12]. Several TFs were manifested to be key regulators such as ANR1 [13], TEOSINTE BRANCHED1/CYCLOIDEA/PROLIFERATING CELL FACTOR1-20 (TCP20) [14], TGA1, and TGA4 [15].

Varala et al. [12] found 172 nitrate-responsive TFs which responded to nitrate supply within 2 h at the messenger RNA (mRNA) level through a transcriptome analysis. To identify key genes involved in NUE in *Arabidopsis*, we explored the transcriptome data of Varala et al. [12], and found that *LATERAL ORGAN BOUNDARY DOMAIN* 39 (*LBD39*), *basic LEUCINE-ZIPPER 8* (*bZIP8*), *LBD38*, and *ETHYLENE-RESPONSIVE FACTOR 56* (*ERF56*) (Figure S1) were on the top of the list of differentially expressed TFs which were ranked according to the maximum absolute log2foldChange across all the time points in the shoot, suggesting that they might be pivotal for nitrate signaling. Among these four genes, little is known about the role of *ERF56*. In addition, *ERF56* has a higher mRNA level in the shoot than in the root (Figures S1 and S2). Thus, it was selected for further analyses.

The APETALA2/ETHYLENE-RESPONSIVE Factor (AP2/ERF) family is a large TF family including 122 members in *A. thaliana*, of which ERF56 (AT2G22200) belongs to the group I (A-6) subfamily [16]. Brooks et al. [17] reported that 5203 genes were putatively targeted by ERF56 using a cell-based method. However, the function of *ERF56* in nitrate signaling remains not yet entirely known. In this research, we confirmed that the transcription of *ERF56* responds to nitrate quickly and revealed that *ERF56* negatively regulates nitrate-dependent plant growth, and that the ERF56 protein is a TF mainly expressed at the region of maturation of roots and in anthers and is involved in the processes of water transport, inorganic molecule transmembrane transport, secondary metabolite biosynthesis, and cell wall organization.

## 2. Results

### 2.1. Transcription of ERF56 Responds Quickly to Nitrate Provision

**Figure 1:**
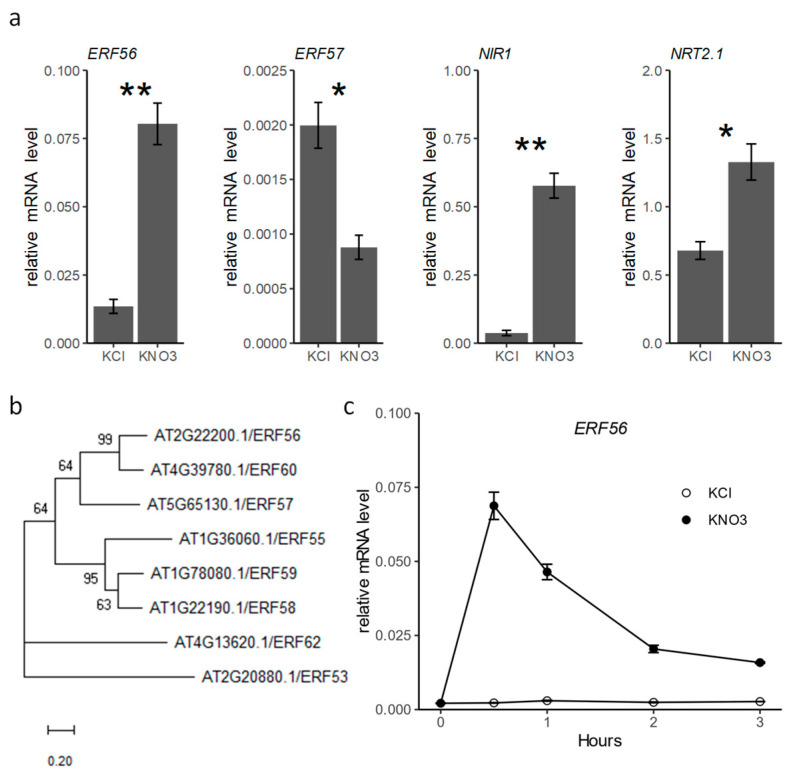
Transcription of *ERF56* is highly induced by NO_3_^−^. (**a**) The relative mRNA levels of *ERF56*, *ERF57*, *NIR1*, and *NRT2.1* in the seedlings of 10-day-old wild-type plants deficient in nitrate treated with 10 mM KNO_3_ or KCl (mock treatment) for 2 h. Values are means ± SE (*n* = 3). ** and * indicate *p* < 0.01 and *p* < 0.05 with Student’s *t*-test, respectively. ACT8 was used as a reference gene. (**b**) A phylogenetic tree of the *ERF56* homologs. (**c**) Dynamic relative mRNA levels of *ERF56* in the seedlings of 10-day-old wild-type plants deficient in nitrate treated with 10 mM KNO_3_ or KCl. Values are means ± SE (*n* = 3).

It was reported that the mRNA expression of *ERF56* is induced early by nitrate to a high level in *Arabidopsis* [12]. Other public data also showed that the mRNA of *ERF56* is quickly induced by nitrate [18,19]. We then inspected the transcription of *ERF56* in response to a nitrate treatment with reverse transcription quantitative PCR (RT-qPCR). It was observed that the mRNA level of *ERF56* is 6.0-fold upregulated after 2 h 10 mM NO_3_^−^ treatment in seedlings deficient in nitrate (Figure 1a). As expected, the transcription of both *NIR1* and *NRT2.1*, two known nitrate-induced genes, is upregulated by the treatment of nitrate (Figure 1a).

ERF56 is a member of the A-6 group of the AP2/ERF family in *Arabidopsis* [16]. Seven homologs were obtained by a blast search with the protein sequence of *ERF56* against the genome database of *Arabidopsis thaliana*, which had a much higher similarity to ERF56 than other proteins. These proteins include all the members of the group I A-6 group of the AP2/ERF family except ERF54 and ERF61 (Figure 1b). The phylogenetic analysis with the alignment of whole protein sequences indicated that ERF60 (AT4G39780) has the closest relationship with ERF56 (Figure 1b), while the analysis based on the alignment of AP2/ERF domain sequences of these proteins demonstrated that ERF57 (AT5G65130) is the closest neighbor of ERF56 [16]. However, expression data of electronic fluorescent pictographs (https://bar.utoronto.ca/eplant/, accessed on 6 October 2022) showed that the mRNA level of *ERF60* is highest in the developing embryo. Thus, we examined the transcription change in *ERF57* in response to NO_3_^−^ treatment in this research. Interestingly, the transcription of *ERF57* is 0.43-fold downregulated after a 2 h NO_3_^−^ provision in contrast to the transcriptional upregulation of *ERF56* by nitrate provision (Figure 1a). It was also observed that the mRNA level of *ERF57* is much lower than that of *ERF56* in seedlings. The mRNA of *ERF57* is 0.15 folds of that of *ERF56* before NO_3_^−^ provision. The higher mRNA level of *ERF56* compared with that of *ERF57* in the seedling might indicate that *ERF56* has a more dominant role than *ERF57* in nitrate signaling.

We further surveyed the dynamic mRNA level of *ERF56* in seedlings in response to nitrate^-^ provision with an independent experiment. The result also confirmed that the transcription of *ERF56* is highly induced by nitrate (Figure 1c). In addition, it was noted that the mRNA of *ERF56* reaches the highest level within 30 min of the 10 mM NO_3_^−^ treatment, and then decreases with time (Figure 1c).

Taken together, the mRNA level of *ERF56* is quickly upregulated to a high level by nitrate provision, which indicates that *ERF56* might take a crucial part in nitrate signaling.

### 2.2. ERF56 Represses Nitrate-Dependent Plant Growth

**Figure 2:**
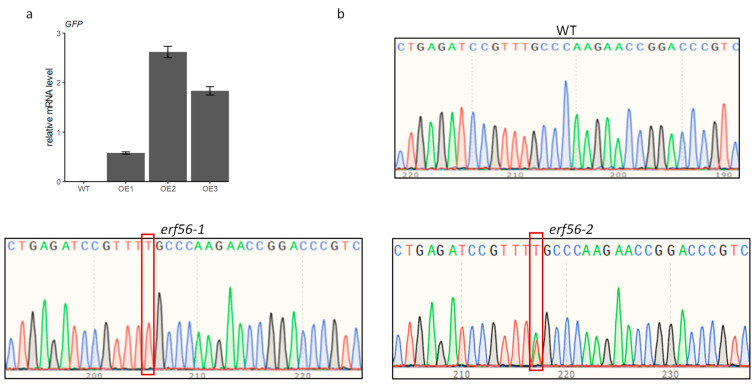
Confirmation of *ERF56* OEs and *erf56* mutants. (**a**) Transcription of *GFP* in leaves of 20-day-old WT (control) and OE T2 plants; in the OEs, the C-terminal of *ERF56* was fused in frame with *GFP*; and values are means ± SE (*n* = 3). *ACT8* was used as the reference gene. (**b**) Mutation sites in *ERF56* of two *erf56* mutants. The red frames indicated the inserted bases.

To uncover the role of *ERF56* in nitrate signaling in *Arabidopis*, transgenic *ERF56* overexpressors (OEs) and *erf56* mutant lines were generated. OE lines were confirmed with RT-qPCR using primers targeting the *GFP*, which is in-frame fused to the C-terminal of the transgenic *ERF56* (Figure 2a). *erf56* mutants were generated by using gene editing based on the CRISPR/Cas9 system. Mutants were confirmed by sequencing. One-base insertions at the 263 bp of the coding sequence (CDS) resulted in frameshift mutations for two independent mutants *erf56-1* and *erf56-2* (Figure 2b).

**Figure 3:**
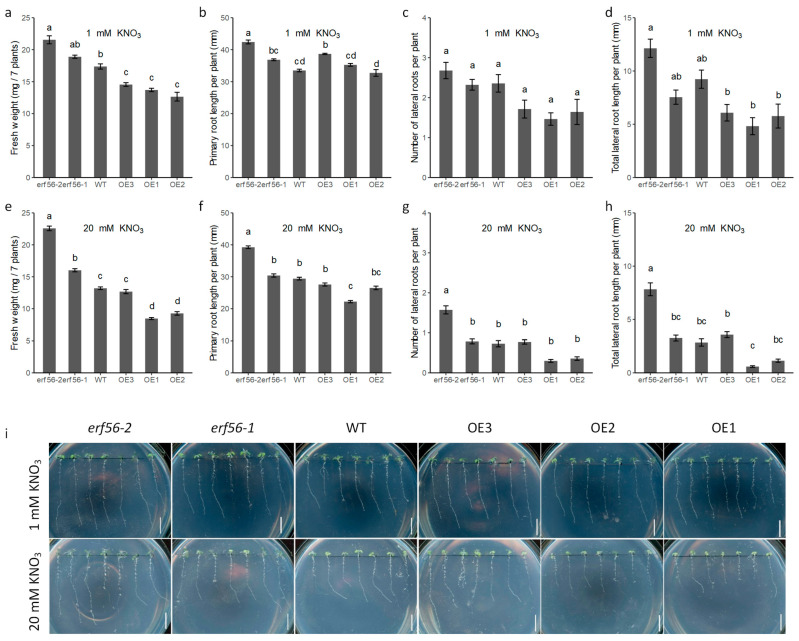
Influence of *ERF56* on plant growth under conditions with different concentrations of KNO_3_. (**a**–**h**) Quantitative analysis of seedlings growth of *ERF56* OEs, *erf56* mutants, and the WT type on the modified 1/2× MS medium containing 1 mM KNO_3_ or 20 mM KNO_3_. Data are means ± SE (*n* = 4 replicates for 1 mM KNO_3_, *n* = 10 replicates for 20 mM KNO_3_, and each replicate has 7 seedlings). Data were analyzed with two-way ANOVA. Multiple comparisons of genotype effects were performed using LSD-test with significant differences being indicated by letters (*p* ≤ 0.05). (**i**) Representative seedlings grown on the modified 1/2× MS medium containing 1 mM KNO_3_ or 20 mM KNO_3_. Bar = 1 cm.

Three *ERF56* OEs and the two *erf56* mutants were grown in Petri dishes with medium containing 1 mM KNO_3_ or 20 mM KNO_3_ along with wild-type plants. The *erf56* mutants had a higher fresh weight (FW) than the wild-type under both conditions, while the OEs had a lower FW than the wild-type (Figure 3a,e,i). The *erf56* mutants demonstrated higher FW-promoting rates under the condition of high nitrate than under the condition of low nitrate, while all the OE lines except OE3 showed higher FW-repressing rates under the condition of high nitrate than under the condition of low nitrate. *erf56-1* and *erf56-2* displayed a 8.7% and 24.0% increase in FW relative to the wild-type on the medium containing the 1 mM KNO_3_ condition, respectively, while they showed a 21.4% and 70.0% increase in FW on the medium containing 20 mM KNO_3_, respectively (Figure 3a,e). The mean FWs of OE1, OE2, and OE3 were 78.8%, 72.8, and 83.7% of that of the wild-type, respectively, on the medium containing 1 mM KNO_3_, while these were 64.1%, 70.2%, and 96.1% of that of the wild-type on the medium containing 20 mM KNO_3_ (Figure 3a,e).

The expression of *ERF56* also repressed the development of both primary roots and lateral roots on the medium containing 1 mM or 20 mM NO_3_^−^ on the whole. The effects of these genotypes on the primary root length (PRL) under the high-nitrate condition agreed well with that on the FW (Figure 3f). However, this result was not observed for the plants grown under the 1 mM NO_3_^−^ condition (Figure 3b). The effects of the genotypes on the number of lateral roots per plant (NLR) and total lateral root length per plant (LRL) under the 1 mM NO_3_^−^ condition were well in line with that on the FW, except *erf56-1* (Figure 3c,d). The severe repression on NLR and LRL by the high nitrate was observed for all the genotypes. These results suggested that *ERF56* negatively regulates plant growth systematically under the condition of the supplementation of nitrate and the effect of *ERF56* on FW is not dependent on its effect on root growth.

Together, the overexpression of *ERF56* represses plant growth, and the knockout of *ERF56* promotes plant growth under the condition of the supplementation of nitrate, and these effects are more obvious under a condition with a high concentration of nitrate.

### 2.3. ERF56 Is a Nucleus-Localized Protein and Is Mainly Expressed in the Maturation Region of Roots and Anthers

**Figure 4:**
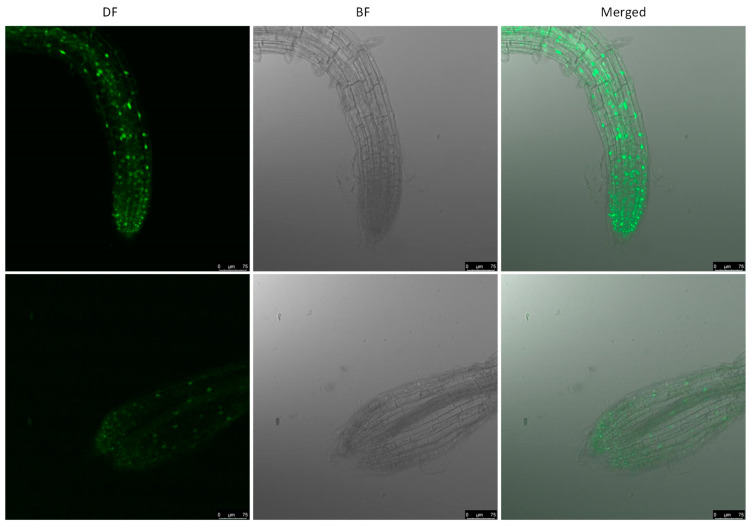
ERF56 protein is localized into nuclei in *Arabidopsis*. Confocal imaging was performed with roots of *35S:ERF56:GFP* transgenic plants. DF, the dark field image with laser-aided confocal laser scanning. BF, the bright field image with transmitted light. The top and bottom panels show the results for two different transformants, respectively.

ERF56 was predicted to be a transcriptional factor [16]. We first performed the prediction of the subcellular localization of the ERF56 protein with Plant-mSubP [20]. The result showed that ERF56 has values of 35%, 34%, and 22% for localization into nuclei, plastid, and cytoplasm, respectively. To verify that ERF56 is a nucleus-localized protein, *ERF56* OE plants which express *GFP* fused to the c-terminal of transgenic *ERF56* were subjected to observation with a laser scanning confocal microscope. Fluorescence signals were detected in the nuclei in these plants (Figure 4), indicating ERF56 is localized into nuclei, which is consistent with the function of a transcriptional factor. This result was further confirmed by the subcellular localization assay with the transient expression of *GFP*-fused *ERF56* in leaves of *N. benthamiana* plants (Figure S3).

**Figure 5:**
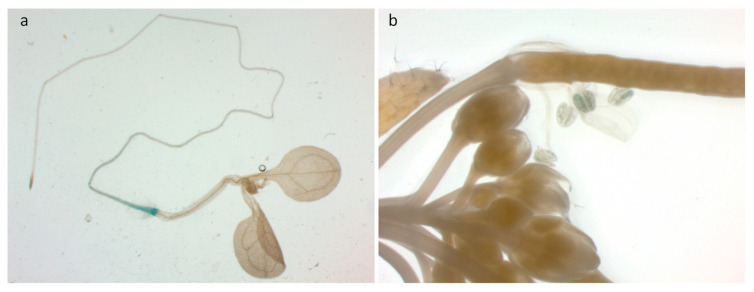
GUS staining images of *proERF:GUS* transgenic plants. (**a**) The image of an 8-day-old seedling. (**b**) The image of an inflorescence of an adult plant.

To examine where ERF56 functions in plants, we generated transgenic *proERF56:GUS* reporter lines in the wild-type. Histochemical glucuronidase (GUS) staining seedlings, as well as leaves, stems, and inflorescences from adult plants of these lines exhibited that the activity of GUS is high in the maturation region of roots (Figure 5a) and anthers (Figure 5b). These data suggested that *ERF56* is mainly expressed in the region of maturation of roots in seedlings. It is interesting that the data of Varala et al. [12] showed the mRNA level of *ERF56* is higher in shoots than in roots in seedlings (Figure S1).

### 2.4. NLP7 Might Not Directly Target the Promoter of ERF56

**Figure 6:**
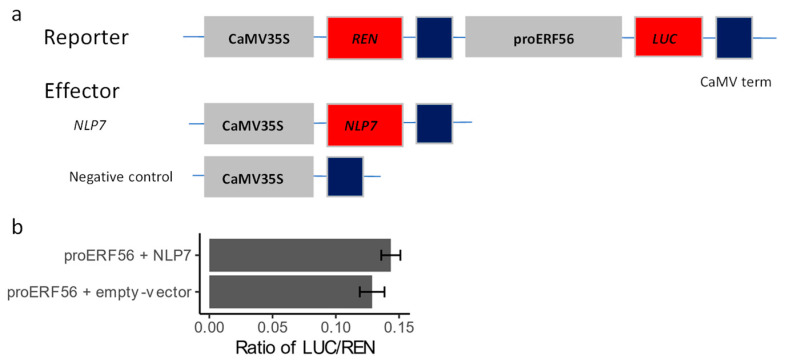
Interaction of NLP7 protein with the promoter of *ERF56*. (**a**) Schematic illustration of constructs used in the Dual-LUC assay. The *35S:REN-proERF56:LUC* reporter plasmid was transiently expressed in the leaves of *Nicotiana benthamiana* plants together with the *35S:NLP7* effector plasmid or empty control plasmid. The reporter plasmid contains an *LUC* gene derived by the promoter of *ERF56 proERF56* to monitor the effect of NLP7 on the *proERF56* and a 35S-derived *REN* used as an internal control. Grey, red and blue boxes indicate sequences of promoters, CDS, CAMV terms, respectively. (**b**) The expression of reporter genes. Data are means ± SE (*n* = 5), and *p* = 0.608 using the Student’s *t*-test.

It was found from the data of Liu et al. [11] that the transcription of *ERF56* is up-regulated by the transient overexpression of *NLP7* in mesophyll protoplasts isolated from wild-type plants. Thus, we investigated whether the transcription of *ERF56* was directly regulated by NLP7 using a dual-luciferase (LUC) assay. A reporter carrying *LUC* driven by the promoter of *ERF56* was co-expressed with an effector carrying *NLP7* driven by CaMV35S in the leaves of *Nicotiana benthamiana*, with *Renilla luciferase* (*REN*) derived by CaM35S used as an internal control (Figure 6a). The results showed that the LUC activity was not significantly altered in the presence of *NLP7* (Figure 6b), suggesting that NLP7 might not play a substantial role in the transcriptional regulation of *ERF56*. This result was in line with the Chip-Seq result [8], in which no NLP7-binding domains were identified around the locus of *ERF56*.

### 2.5. Genes Targeted by ERF56

We generated transgenic lines, in which the overexpression of *ERF56* can be induced by ß-estradiol to avoid finding many indirect targets in experiments, to identify ERF56-targeted genes by disturbing the expression of *ERF56*. Transcriptome profiling of an inducible *ERF56* overexpressor *ER8-ERF56-1* in response to ß-estradiol was conducted. A principal component analysis of gene counts showed that the samples of ß-estradiol-treated *ER8-ERF56-1* plants were clearly distinguished from the samples of mock-treated *ER8-ERF56-1* plants and the samples of ß-estradiol-treated or mock-treated WT plants (Figure S4), suggesting that these data can be used to discover differentially expressed genes (DEGs) due to the ß-estradiol-induced overexpression of *ERF56*.

**Figure 7:**
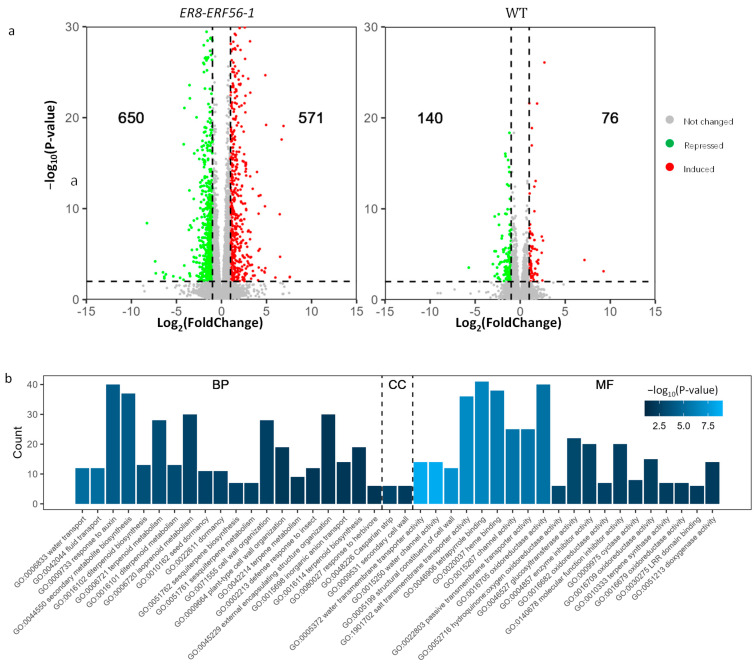
Identification of genes targeted by ERF56. (**a**) Volcano plots of log2 fold changes and negative log10 P-values of gene expression in the inducible *ERF56* OE line *ER8-ERF56-1* and WT after 3 h ß-estradiol treatments, respectively. The 10-day-old plants deficient in nitrate were transferred to a fresh nitrate-free medium either containing 2 uM 17-ß-estradiol or not, as a mock treatment (MOCK), for 3 h. Three biological replicates, each of which had 20 plants, were used for the transcriptome analysis. DEGs were determined using *p*-value ≤ 0.01 and |log_2_fold change| ≥ 1, with up-regulated genes in samples treated with ß-estradiol being plotted as red points, down-regulated genes being plotted as green points, and the others being plotted as grey points. (**b**) Top over-presented GO terms in biological process (BP), cellular component (CC), and the molecular function (MF) for the 1038 candidate genes targeted by ERF56. The 1038 candidate genes were selected from 1221 DEGs in *ER8-ERF56-1*, which were not differentially expressed in the WT in response to ß-estradiol (*p*-value ≤ 0.01) and did not include *ERF56.* A list of each of the top 20 significantly enriched terms in BP and MF, and the only 2 enriched in CC selected with the lowest P-value and the number of genes mapped within each term are shown, with enriched terms being determined by *p*-value ≤ 0.05.

A total of 1221 genes were differentially expressed in *ER8-ER56-1* in response to the ß-estradiol treatment (|log_2_FoldChange| ≥ 1 and *p*-value ≤ 0.01), while 216 genes were differentially expressed in the wild-type in response to the ß-estradiol treatment (Figure 7a). Among the 1221 DEGs, 1039 genes were not differentially expressed between the ß-estradiol and mock treatment in the WT (determined under the threshold *p*-value ≤ 0.01), including *ERF56* which was induced by ß-estradiol in *ER8-ER56-1*, as expected. To be conservative, these 1038 genes excluding *ERF56* were considered as candidate direct targets of the ERF56 protein (Table S1). A total of 228 of the 1038 genes were present in the DEGs obtained in the TARGET (Transient Assay Reporting Genome-wide Effects of Transcription factors) assay of the *ERF56* [17], with an enrichment *p*-value = 0.02, and 87 genes existed in the DEGs gained in the wild-type in response to the nitrate treatment [6], with an enrichment *p*-value < 10^−5^, indicating that the nitrate-regulated genes were over-represented in these 1038 candidate direct targets of ERF56.

The over-representation analysis of these candidate targets of ERF56 demonstrated that 69 gene ontology (GO) terms were significantly enriched (Table S2). Each of the top 20 enriched GO terms in the biological process (BP) and the molecular function (MF), and the only 2 enriched terms in the cellular component (CC) are listed in Figure 7b. The water transport, secondary metabolic process, response to auxin, dormancy process, and cell wall organization are severely disturbed by the overexpression of *ERF56* (Figure 7b). Li et al. [21] reported that ERF55 and ERF58, two members of the group 1 subfamily of AP2/ERF TFs, are involved in regulating the dormancy of *Arabidopsis* seeds. Thus, it was not surprising that the overexpression of *ERF56* affected the transcription of genes in the dormancy process in our research due to the close evolutional relationship between *ERF56*, *ERF55*, and *ERF58* (Figure 1b).

It was intriguing that several processes of transmembrane transports were over-presented, including water transport (GO:0006833), inorganic anion transport (GO:0015698), inorganic ion transmembrane transport (GO:0098660), nitrate import (GO:1902025), and zinc ion transmembrane transport (GO:0071577) (Figure 7b, Table S2). In addition, the process of the response to nutrient levels (GO:0031667) was also over-presented (Figure 7b, Table S2). Water transport was the most enriched term of BP (Figure 7b). Among the 39 expressed water transporter-coding genes, 12 were significantly differentially expressed between treatments in *ER8-ERF56-1*, all of which were up-regulated by the overexpression of *ERF5* (Table S3).

In the nitrate import process (Table S3), the transcription of *NRT1.11* (*AT1G52190*), *NRT2.4* (*AT5G60770*), and *AT5G66816* was downregulated by the overexpression of *ERF56*. NRT1.11 plays a role in the xylem-to-phloem transport of nitrate for redistributing xylem-borne nitrate under high-nitrate conditions [22]. NRT2.4 participates in nitrate transport under the condition of nitrogen starvation [23]. The mRNA of *NRT1.7* (*AT1G69870*), *NRT2.7* (*AT5G14570*), and *CEPR2* (*AT1G72180*) were up-regulated by the overexpression of *ERF56*. *NRT1.7* encodes a nitrate transporter responsible for the remobilization of nitrate from older leaves into younger leaves [24]. *NRT2.7* encodes a nitrate transporter that controls nitrate accumulation in seeds [25]. The transcription of *AMT1;3* and *AMT1;5* was repressed by the overexpression of *ERF56* (Table S3). AMT1;3 (AT3G24300) and AMT1;5 (AT3G24290) are two ammonium transporters contributing to ammonium uptake in *Arabidopsis* [26,27].

Among genes mapped to the process of inorganic anion transport (Table S3), three encode the phosphate transporter, including *PHT1;1* (*AT5G43350*), *PHT1;2* (*AT5G43370*), and *PHT5;3* (*AT4G22990*). The transcription of *PHT1;1* and *PHT1;2* was repressed by the overexpression of *ERF56*, while the transcription of *PHT5;3* was induced. PHT1;1 and PHT1;2 are two of the four main phosphate transporters for Pi uptake in *Arabidopsis* [28,29,30]. PHT5;3 contributes to cytosol-to-vacuole Pi partitioning [31]. These data suggested that the expression of *ERF56* might repress the uptake of phosphate and decrease the level of cytosolic Pi. Among zinc transporter-coding genes [32], the transcription of *AT1G05300*, *AT2G32270*, *AT1G60960*, and *AT4G33020* were repressed by the overexpression of *ERF56*, while that of *AT1G31260* was induced (Table S3). *AT1G31260* was expressed at a low level. These data indicated that the overexpression of *ERF56* might repress the uptake of zinc or Fe.

Secondary metabolite biosynthesis was the fourth over-represented process (Figure 7b). It was intriguing that several related metabolic processes were enriched, such as the diterpenoid biosynthetic process (GO:0016102), terpenoid metabolic process (GO:0006721), diterpenoid metabolic process (GO:0016101), isoprenoid metabolic process (GO:0006720), and sesquiterpene biosynthetic process (GO:0051762) (Figure 7b). Gibberellins (GAs) are a class of tetracyclic diterpenoid carboxylic acids, some members of which act as plant hormones. Among the 13 genes mapped to the diterpenoid biosynthetic process, the transcription of 10 genes was repressed by the overexpression of *ERF56*, including three gibberellin oxidase-coding genes, *GA3ox1* (*AT1G15550*), *GA3ox2* (*AT1G80340*), and *GA20ox2* (*AT5G51810*) (Table S4). Another gibberellin oxidase-coding gene *GA2ox1* (*AT1G78440*) was upregulated at the mRNA level. GA catabolism is necessary for keeping the bioactivity homeostasis of GA in plants. GA3ox1, GA3ox2, and GA20ox2 are central regulators of the GA interaction network [33]. These data implied that *ERF56* might affect plant growth through regulating active GA levels. The auxin responsive process was the third enriched term of BP (Figure 7b), indicating that the overexpression of *ERF56* might adjust the distribution of auxin in plants.

The process of cell wall organization (GO:0071555) was another over-represented term of BP (Figure 7b). Several genes encoding expansins or cell wall architecture-related proteins were differentially expressed in response to the ß-estradiol treatment in *ER8-ERF56-1* (Table S5), which included four genes encoding expansins, four genes encoding proteins of the Plant Invertase/Pectin Methylesterase Inhibitor Superfamily (INV/PMEI), four genes encoding pectin lyase-like proteins, and eight genes encoding proteins of the proline-rich extensin-like family. ß-expansins contribute to PH-dependent cell-wall loosening to promote cell elongation and other developmental events [34]. Pectin is a major component of the primary cell wall [35]. INV/PMEI includes INVs catalyzing the cleavage of sucrose into glucose and fructose, PMEs controlling pectin methylesterification, as well as INVI and PMEI regulating the activities of specific INV/PME proteins [36]. The demethylation of pectin makes it more susceptible to pectin-degrading enzymes, such as polygalacturonases (PGs) and pectate lyases (PLs) [37,38]. Extensins are plant cell wall hydroxyproline-rich glycoproteins, which affect cell wall reinforcement in higher plants [39]. Thus, these data suggested that ERF56 participates in the modulation of cell wall architecture to regulate plant growth.

### 2.6. Candidate Cis-Regulatory Elements Targeted by ERF56

**Figure 8:**
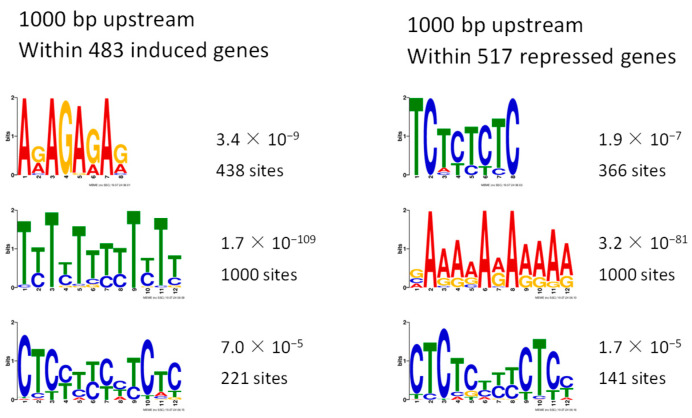
Identification of cis-regulatory elements putatively targeted by ERF56. Weight matrix representation of the motifs was retrieved using the soft MEME from 1000-bp promoter sequences upstream the start codons of the 483 induced and 517 repressed nuclear genes regulated by ERF56, respectively.

To determine candidate cis-regulatory elements bound by ERF56, 1000-bp sequences upstream start codons were extracted from the database for both the 483 ERF56-induced and 517 ERF56-repressed nuclear genes among the 1038 DEGs. These sequences were retrieved for conserved motifs with Multiple EM for Motif Elicitation (MEME) [40]. Three motifs were found in the promotors of both induced genes and repressed genes under the threshold of E-value < 0.01 (Figure 8). Whether these motifs are genuine sites targeted by ERF56 needs to be confirmed by further experiments.

## 3. Discussion

In this study, we confirmed that the transcription of *ERF56* is quickly induced by nitrate, found that *ERF56* negatively contributes to nitrate-dependent plant growth, and found that ERF56 is a nucleus-localized protein which is highly expressed at the region of maturation of roots and in anthers. Our data also demonstrated possible roles of *ERF56* in nitrate signaling.

The result that *ERF56* is mainly expressed in roots, especially at the region of maturation of roots, at the seedling stage does not agree with the data of Varala et al. [12], which showed *ERF56* had a higher mRNA level in shoots than in roots (Figure S1). Many transcription start sites (TSSs) overlap with start codons [41] and cis-elements of promoters can exist downstream TSSs [42]. Thus, whether *ERF56* has cis-elements in regions other than the scrutinized 1.5 Kb sequence needs to be investigated in the future.

NLP7 is a pivotal member of NLPs [7,8,10]. In this research, no significant NLP7-regulating effect on the activity of the 1.5 Kb promoter sequence of *ERF56* was perceived in the dual-luciferase assays (Figure 6b). Also, no NLP7-binding signals were observed around the locus of *ERF56* in the CHIP-chip assay [8]. In addition, Konishi et al. [9] showed the effect of the *nlp2* mutant on the transcript of *ERF56* was stronger than that of the *nlp7* mutant. These results suggested that nitrate regulates the transcription of *ERF56* mainly through NLP2. However, it was noted that the transcription of *ERF56* is up-regulated by the transient overexpression of *NLP7* [11]. We suspected it was the receptor role of NLP7 that resulted in the upregulation of *ERF56* in the experiment. Further evidence is needed to confirm the hypothesis for the relationships of *ERF56* with NLP2 and NLP7.

The transcriptome analysis demonstrated that ERF56 might target genes of the processes of water transport, inorganic molecule transmembrane transport, secondary metabolite biosynthesis, cell wall organization, dormancy, and defense response to insects or herbivores. Water transport was the most enriched term of BP. But none of these genes were significantly differentially expressed between the nitrate treatment and the control in the WT in the public data GSE73437. Water transport contributes to regulating reactive oxygen species (ROS) homeostasis [43,44,45]. ROS are major regulatory molecules in plants [45]. The role of the regulation of the water transport process by ERF56 needs to be answered by further experiments.

Nine genes associated with the process of nitrate or ammonium transport were found to be candidate regulation targets of ERF56, six of which were differentially expressed in response to the nitrate treatment in the WT in the public data GSE73437, denoting that *ERF56* participates in the regulation of nitrate/ammonium transmembrane transporting. However, the modes of the regulation of these processes by nitrate remain to be unveiled. Furthermore, genes mapped to the transport processes of nutrients other than nitrogen were also enriched in the candidate targets of ERF56, implying that ERF56 might be a hub site for different nutrient signaling.

Terpenoid metabolism-related processes, especially GA biosynthesis, were disturbed by the overexpression of *ERF56*. GA is a hormone essential for many developmental processes of plants [46]. Thus, it is likely that *ERF56* regulates plant growth by altering the GA level in plants. Phenotypes of OEs and *erf56* mutants demonstrated that *ERF56* indeed regulated plant growth. However, the result showed that *ERF56* is mainly expressed in the roots of seedlings, which means that GA should be moved in plants. Research showed that GA movement is necessary for plant development [46]. It is needed to investigate in future whether the overexpression of *ERF56* decreases the active GA level.

Based on the information mentioned above, we propose a model for *ERF56*-regulating nitrate-dependent plant growth: (1) nitrate modulates the transcription of *ERF56* through NLP2 and (2) *ERF56* directly regulates the uptake and redistribution of inorganic nutrients and GA synthesis in roots. We think it is valuable to examine the effects of *ERF56* homologs on NUE in crops in future.

## 4. Materials and Methods

### 4.1. Plant Material

*A. thaliana* ecotype Columbia-0 (Col-0) was used as the WT. Mutant lines *erf56-1* and *erf56-2*, *ERF56* overexpressors OE1, OE2, and OE3, inducible *ERF56* overexpressor *ER8-ERF56-1*, and transgenic line *proERF56:GUS* lines were used in this study.

Mutant line *erf56-1* and *erf56-2* were obtained by CRISPR/Cas9 technology. A pair of single-guide RNA sequences (sgRNA) were designed to target the CDS of *ERF56* (from 260 bp to 279 bp) and the primers including the sgRNA were cloned into the vector pHEE401E [47]. The resulting construct was used to transform the WT via *Agrobacterium tumefaciens*-mediated floral transformation by the floral dip method [48]. *Cas9*-free mutants were selected from T_2_ plants.

To obtain *ERF56* OEs, the CDS of *ERF56* was amplified from mRNA isolated from the WT using reverse transcription PCR (RT-PCR) and cloned into the NcoI site of a modified version of the vector pCAMBIA1305. The C-terminal of *ERF56* was fused in frame with *GFP*. To obtain inducible *ERF56* overexpressing lines, the amplified CDS of *ERF56* was cloned into the MCS of the vector pER8. The WT was used for transformation and seeds from T_2_ homozygous transgenic plants were used in the study. To obtain *proERF56:GUS* reporter lines, the genomic sequence 1.5 Kb upstream the start codon of *ERF56* was amplified from the DNA of WT plants and cloned into the NcoI site of the vector pCAMBIA3301 to replace the original *35S* promoter. All primers used for constructing these plasmids are listed in Table S6.

### 4.2. Genotyping of Erf56 Mutants

Primers near the sequence of *ERF56* targeted by the guide RNA were designed. Genomic DNA was extracted from plants’ leaves for PCR with these primers and Vazyme Phanta Flash Master Mix (Vazyme, Nanjing, China). PCR amplicons were sequenced to identify deletions, insertions, or point mutations. PCRs with *Cas9*-specific and *hyg*-specific primers were performed to identify *Cas9*-free mutants. The used primers are listed in Table S6.

### 4.3. Growth Condition and Treatments

*Arabidopsis* seeds were sterilized with 70% alcohol for 5 min and a 1:20 dilution of sodium hypochlorite for 5 min, and then washed six times, sown on the surface of solid 1/2× MS or nitrogen-free 1/2× MS basal salt medium supplemented with 1 mM NH_4_Cl and the needed concentration of KNO_3_, 1% sucrose, and 0.8% (*w*/*v*) agar (adjusted to pH 6.0 with NaOH) in petri dishes. Petri dishes with the sown seeds were kept at 4 °C for 2 days in darkness and then placed vertically for the needed days in day/night cycles (16/8 h) at 22 °C. For studying the nitrate-induced transcription of *ERF56*, plants were grown on solidified nitrate-free medium (1/2× MS basal salt medium supplemented with 1 mM NH4Cl) for 5 days, and then transferred to dishes with fresh solidified nitrate-free medium for another 5 days. Then, these plants were transferred to the fresh nitrate-free medium supplemented with 10 mM KNO_3_ or 10 mM KCl, as a mock treatment, for 0.5, 1, 2, or 3 h (s). For phenotyping, plants were grown on solid nitrate-free medium supplied with 1 mM or 20 mM KNO_3_ for 5 days and then transferred to corresponding fresh medium to grow for another 4 days. To grow plants for a long time, the seeds were germinated and then the germinated seedlings were grown on a 1/2× MS medium for 10 days. After that, the seedlings were transferred to soil to grow in an environment-controlled room at 22 °C.

### 4.4. Root Growth Measurements

Petri dishes with plants were scanned with Perfection V550 Photo (EPSON, Beijing, China) using the professional mode with the resolution of 600 DPI and 24-bit-Color image type and with the other parameters being set to default. The root length was measured by using the segmented line selection tool in imageJ V1.54g [49].

### 4.5. In Silico Analysis

Normalized gene counts were downloaded from https://datadryad.org/stash/dataset/doi:10.5061/dryad.248g184 (accessed on 6 October 2022) [12] and were used to calculate the Log_2_FoldChange in each gene at all the time points. DEGs were ranked according to the maximum of the absolute values of the log_2_FoldChange in each gene across all the time points.

### 4.6. Phylogenetic Analysis

The protein sequence of ERF56 was used to retrieve homologs in the *A. thaliana* database. The top seven hits with a much higher similarity to ERF56 than the others were used for the phylogenetic analysis. Protein sequences were aligned with ClustalW using the default parameters, the phylogenetic tree was constructed with the maximum likelihood method, and the test of the branches was calculated based on 1000 replications of bootstraps. Analyses were performed using MEGAX V10.0.5 [50].

### 4.7. RNA Extraction, Reverse Transcription, and Quantitative PCR

The total plants were harvested for RNA extraction. The RNA was prepared using a Aidlab’s plant RNA extraction kit RN09 (Aidlab, Beijing, China) and reverse transcribed to cDNA using the Accurate Biology’s RT Kit (Accurate Biology, Changsha, China). qPCR was performed with a SYBR Green Real-Time PCR Master Mix (Accurate Biology, Changsha, China) using The Applied Biosystems 7500 Real-Time PCR System (ThermFisher Scientific, Waltham, MA, USA) following the comparative CT experiment protocol of the manufacturer. Experiments were performed with three independent RNA samples and three technical replicates. *AC8* was used as the reference gene. The relative gene expression was calculated as described by Livak and Schmittgen [51]. Primers used for qPCRs are listed in Table S6.

### 4.8. Transcriptome Analysis

Ten-day-old plants grown on solidified nitrate-free medium were transferred to nitrate-free medium either containing 2 uM 17-ß-estradiol or not, as a mock treatment, for 3 h. Then, the whole plants were harvested and subjected to RNA extraction. Three biological replicates, each of which contains 20 plants, were used for the transcriptome analysis.

The sequencing was performed at Novogene (Tianjin, China). The mRNA was purified from the total RNA using poly-T oligo-attached magnetic beads and fragmented using divalent cations under an elevated temperature in First-Strand Synthesis Reaction Buffer. First-strand cDNA was synthesized using a random hexamer primer and M-MuLV Reverse Transcriptase (RNase H-). Second-strand cDNA synthesis was subsequently performed using DNA Polymerase I and RNase H. The remaining overhangs were converted into blunt ends via exonuclease/polymerase activities. After the adenylation of 3′ ends of DNA fragments, adaptors were ligated to prepare for hybridization. The library fragments were purified with the AMPure XP system in order to select cDNA fragments of preferentially 370~420 bp in length. Then, PCR was performed with Phusion High-Fidelity DNA polymerase, Universal PCR primers, and the Index Primer. At last, the PCR products were purified.

After the library quality control, different libraries were pooled based on the effective concentration and targeted data amount. The 5′ end of each library was phosphorylated and cyclized. Subsequently, loop amplification was performed to generate DNA nanoballs. These DNA nanoballs were finally loaded into the flowcell with DNBSEQ-T7 for sequencing.

Clean reads were obtained after removing the reads of low quality, reads containing adapter contamination, and adapter sequences of RAW data with the soft fastp V0.24.0 [52]. The clean reads were aligned to the genome sequence (TAIR10) using Hisat2 V2.0.1-beta [53], and assembled using stringtie V1.2.1 [54]. DEGs were determined using the R package DESeq2 V1.42.1 [55]. The GO over-representation analysis was implemented by applying the R package clusterProfiler V4.0 [56].

### 4.9. Histochemical GUS Staining and Confocal Microscopy Analysis

The seeds were germinated, and next, the germinated seedlings were grown on solidified 1/2× MS medium for 8 days to prepare the seedling samples for histochemical GUS staining. Plants grown in the soil till flowering were used to prepare the adult plant samples for GUS staining. The harvested samples were stained using the GUS stain kit SL7160 (Coolaber, Beijing, China) at 37 °C in the dark for 24 h. After staining, the samples were transferred to small Petri dishes containing a 70% ethanol solution and incubated at room temperature until the leaves of the control were white, with the solution being renewed every 10 min. Images were captured using a Zeiss SteREO Discovery V12 microscope (Zeiss, Oberkochen, Germany).

For the in planta GFP analysis, OE1 and OE3 seedlings, which express in-frame fused *GFP*, were grown on solidified nitrate-free medium for 5 days. Then, the roots of the seedlings were observed and imaged using a Leica SP8 confocal laser scanning microscope (Leica, Wetzlar, Germany). GFP was excited using light of a 488 nm wavelength, and the emitted light was captured at 505–555 nm. For the subcellular localization of ERF56 with a transient expression assay, the leaves of 30-day-old *N. benthamiana* were infiltrated with *Agrobacteria* containing the plastid carrying *ERF56*, which were used to generate transgenic OEs or the empty control plastid. The infiltrated *N. benthamiana* plants were incubated at 24 °C for 36 h. Then, epidermal cells of their leaves were subjected to confocal imaging.

### 4.10. Dual-Luciferase Assays

To generate the reporter plasmid, the 1.5 Kb genomic sequences upstream the start codon of *ERF56* of the WT were cloned into the vector pGreenII0800-LUC to derive the expression of *LUC.* The CDS of *NLP7* was amplified from cDNA derived from the WT and cloned into the vector pGreenII 62-SK to generate a 35S-derived effector plasmid. *N. benthamiana* leaves were co-infiltrated with *Agrobacteria* containing the reporter plasmid, and *Agrobacteria* containing the effector plasmid or the empty pGreenII 62-SK plasmid (as a control). The infiltrated *N. benthamiana* were incubated at 24°C for 36 h, and then, 8 mm leaf discs around the points of infiltration were harvested. The LUC activity and REN activity were determined using a Dual-Luciferase Reporter Gene Assay Kit (Yeasen, Shanghai, China) on the Tecan M200 system (Tecan, Grödig, Austria). Each sample was measured three times.

### 4.11. Cis-Regulatory Element Discovery

The 1000-bp sequences upstream start codons of genes were extracted from the genome (TAIR10) to identify candidate cis-regulatory elements with MEME Suit 5.5.7 [40]. Two modes of MEME, Any Number of Repetitions (ANR) and Zero or One Occurrence per Sequence (ZOOPS), were used in the study. For ANR, the used parameters were -nmotifs30-minw6-maxw12-revcomp-dna. For ZOOPs, the used parameters were -nmotifs30-minw6-maxw8-revcomp-dna.

## Data Availability

The raw data of RNA-seq are available at https://ngdc.cncb.ac.cn database under the project ID of PRJCA031029 (accessed on 11 January 2025).

## Supplementary Materials

The following supporting information can be downloaded at: https://www.mdpi.com/article/10.3390/ijms26020613/s1.

## References

[1] Raun W.R., Johnson G.V. (1999). Improving Nitrogen Use Efficiency for Cereal Production. Agron. J..

[2] Dechorgnat J., Nguyen C.T., Armengaud P., Jossier M., Diatloff E., Filleur S., Daniel-Vedele F. (2011). From the Soil to the Seeds: The Long Journey of Nitrate in Plants. J. Exp. Bot..

[3] Tsay Y.F., Schroeder J.I., Feldmann K.A., Crawford N.M. (1993). The Herbicide Sensitivity Gene *CHL1* of Arabidopsis Encodes a Nitrate-Inducible Nitrate Transporter. Cell.

[4] Ho C.-H., Lin S.-H., Hu H.-C., Tsay Y.-F. (2009). CHL1 Functions as a Nitrate Sensor in Plants. Cell.

[5] Riveras E., Alvarez J.M., Vidal E.A., Oses C., Vega A., Gutiérrez R.A. (2015). The Calcium Ion Is a Second Messenger in the Nitrate Signaling Pathway of Arabidopsis. Plant Physiol..

[6] Liu K.-H., Niu Y., Konishi M., Wu Y., Du H., Sun Chung H., Li L., Boudsocq M., McCormack M., Maekawa S. (2017). Discovery of Nitrate-CPK-NLP Signalling in Central Nutrient-Growth Networks. Nature.

[7] Konishi M., Yanagisawa S. (2013). Arabidopsis NIN-like Transcription Factors Have a Central Role in Nitrate Signalling. Nat. Commun..

[8] Marchive C., Roudier F., Castaings L., Bréhaut V., Blondet E., Colot V., Meyer C., Krapp A. (2013). Nuclear Retention of the Transcription Factor NLP7 Orchestrates the Early Response to Nitrate in Plants. Nat. Commun..

[9] Konishi M., Okitsu T., Yanagisawa S. (2021). Nitrate-Responsive NIN-like Protein Transcription Factors Perform Unique and Redundant Roles in Arabidopsis. J. Exp. Bot..

[10] Castaings L., Camargo A., Pocholle D., Gaudon V., Texier Y., Boutet-Mercey S., Taconnat L., Renou J.-P., Daniel-Vedele F., Fernandez E. (2009). The Nodule Inception-like Protein 7 Modulates Nitrate Sensing and Metabolism in Arabidopsis. Plant J. Cell Mol. Biol..

[11] Liu K.-H., Liu M., Lin Z., Wang Z.-F., Chen B., Liu C., Guo A., Konishi M., Yanagisawa S., Wagner G. (2022). NIN-like Protein 7 Transcription Factor Is a Plant Nitrate Sensor. Science.

[12] Varala K., Marshall-Colón A., Cirrone J., Brooks M.D., Pasquino A.V., Léran S., Mittal S., Rock T.M., Edwards M.B., Kim G.J. (2018). Temporal Transcriptional Logic of Dynamic Regulatory Networks Underlying Nitrogen Signaling and Use in Plants. Proc. Natl. Acad. Sci. USA.

[13] Zhang H., Forde B.G. (1998). An Arabidopsis MADS Box Gene That Controls Nutrient-Induced Changes in Root Architecture. Science.

[14] Guan P., Wang R., Nacry P., Breton G., Kay S.A., Pruneda-Paz J.L., Davani A., Crawford N.M. (2014). Nitrate Foraging by Arabidopsis Roots Is Mediated by the Transcription Factor TCP20 through the Systemic Signaling Pathway. Proc. Natl. Acad. Sci. USA.

[15] Alvarez J.M., Riveras E., Vidal E.A., Gras D.E., Contreras-López O., Tamayo K.P., Aceituno F., Gómez I., Ruffel S., Lejay L. (2014). Systems Approach Identifies TGA1 and TGA4 Transcription Factors as Important Regulatory Components of the Nitrate Response of Arabidopsis Thaliana Roots. Plant J. Cell Mol. Biol..

[16] Nakano T., Suzuki K., Fujimura T., Shinshi H. (2006). Genome-Wide Analysis of the *ERF* Gene Family in Arabidopsis and Rice. Plant Physiol..

[17] Brooks M.D., Cirrone J., Pasquino A.V., Alvarez J.M., Swift J., Mittal S., Juang C.-L., Varala K., Gutiérrez R.A., Krouk G. (2019). Network Walking Charts Transcriptional Dynamics of Nitrogen Signaling by Integrating Validated and Predicted Genome-Wide Interactions. Nat. Commun..

[18] Scheible W.-R., Morcuende R., Czechowski T., Fritz C., Osuna D., Palacios-Rojas N., Schindelasch D., Thimm O., Udvardi M.K., Stitt M. (2004). Genome-Wide Reprogramming of Primary and Secondary Metabolism, Protein Synthesis, Cellular Growth Processes, and the Regulatory Infrastructure of Arabidopsis in Response to Nitrogen. Plant Physiol..

[19] Wang R., Okamoto M., Xing X., Crawford N.M. (2003). Microarray Analysis of the Nitrate Response in Arabidopsis Roots and Shoots Reveals over 1,000 Rapidly Responding Genes and New Linkages to Glucose, Trehalose-6-Phosphate, Iron, and Sulfate Metabolism. Plant Physiol..

[20] Sahu S.S., Loaiza C.D., Kaundal R. (2020). Plant-mSubP: A Computational Framework for the Prediction of Single- and Multi-Target Protein Subcellular Localization Using Integrated Machine-Learning Approaches. AoB Plants.

[21] Li Z., Sheerin D.J., von Roepenack-Lahaye E., Stahl M., Hiltbrunner A. (2022). The Phytochrome Interacting Proteins ERF55 and ERF58 Repress Light-Induced Seed Germination in Arabidopsis Thaliana. Nat. Commun..

[22] Hsu P.-K., Tsay Y.-F. (2013). Two Phloem Nitrate Transporters, NRT1.11 and NRT1.12, Are Important for Redistributing Xylem-Borne Nitrate to Enhance Plant Growth. Plant Physiol..

[23] Kiba T., Feria-Bourrellier A.-B., Lafouge F., Lezhneva L., Boutet-Mercey S., Orsel M., Bréhaut V., Miller A., Daniel-Vedele F., Sakakibara H. (2012). The Arabidopsis Nitrate Transporter NRT2.4 Plays a Double Role in Roots and Shoots of Nitrogen-Starved Plants. Plant Cell.

[24] Fan S.-C., Lin C.-S., Hsu P.-K., Lin S.-H., Tsay Y.-F. (2009). The Arabidopsis Nitrate Transporter NRT1.7, Expressed in Phloem, Is Responsible for Source-to-Sink Remobilization of Nitrate. Plant Cell.

[25] Chopin F., Orsel M., Dorbe M.-F., Chardon F., Truong H.-N., Miller A.J., Krapp A., Daniel-Vedele F. (2007). The Arabidopsis ATNRT2.7 Nitrate Transporter Controls Nitrate Content in Seeds. Plant Cell.

[26] Loqué D., Yuan L., Kojima S., Gojon A., Wirth J., Gazzarrini S., Ishiyama K., Takahashi H., von Wirén N. (2006). Additive Contribution of AMT1;1 and AMT1;3 to High-Affinity Ammonium Uptake across the Plasma Membrane of Nitrogen-Deficient Arabidopsis Roots. Plant J. Cell Mol. Biol..

[27] Yuan L., Loqué D., Kojima S., Rauch S., Ishiyama K., Inoue E., Takahashi H., von Wirén N. (2007). The Organization of High-Affinity Ammonium Uptake in Arabidopsis Roots Depends on the Spatial Arrangement and Biochemical Properties of AMT1-Type Transporters. Plant Cell.

[28] Ayadi A., David P., Arrighi J.-F., Chiarenza S., Thibaud M.-C., Nussaume L., Marin E. (2015). Reducing the Genetic Redundancy of Arabidopsis PHOSPHATE TRANSPORTER1 Transporters to Study Phosphate Uptake and Signaling. Plant Physiol..

[29] Chien P.-S., Chao Y.-T., Chou C.-H., Hsu Y.-Y., Chiang S.-F., Tung C.-W., Chiou T.-J. (2022). Phosphate Transporter PHT1;1 Is a Key Determinant of Phosphorus Acquisition in Arabidopsis Natural Accessions. Plant Physiol..

[30] Tanaka M., Wallace I.S., Takano J., Roberts D.M., Fujiwara T. (2008). NIP6; 1 Is a Boric Acid Channel for Preferential Transport of Boron to Growing Shoot Tissues in Arabidopsis. Plant Cell.

[31] Luan M., Zhao F., Han X., Sun G., Yang Y., Liu J., Shi J., Fu A., Lan W., Luan S. (2019). Vacuolar Phosphate Transporters Contribute to Systemic Phosphate Homeostasis Vital for Reproductive Development in Arabidopsis. Plant Physiol..

[32] Stanton C., Sanders D., Krämer U., Podar D. (2022). Zinc in Plants: Integrating Homeostasis and Biofortification. Mol. Plant.

[33] Wang S., Wang Y. (2022). Harnessing Hormone Gibberellin Knowledge for Plant Height Regulation. Plant Cell Rep..

[34] Sampedro J., Cosgrove D.J. (2005). The Expansin Superfamily. Genome Biol..

[35] Cosgrove D.J. (2005). Growth of the Plant Cell Wall. Nat. Rev. Mol. Cell Biol..

[36] Coculo D., Lionetti V. (2022). The Plant Invertase/Pectin Methylesterase Inhibitor Superfamily. Front. Plant Sci..

[37] Minic Z., Jouanin L. (2006). Plant Glycoside Hydrolases Involved in Cell Wall Polysaccharide Degradation. Plant Physiol. Biochem. PPB.

[38] Pelloux J., Rustérucci C., Mellerowicz E.J. (2007). New Insights into Pectin Methylesterase Structure and Function. Trends Plant Sci..

[39] Castilleux R., Plancot B., Vicré M., Nguema-Ona E., Driouich A. (2021). Extensin, an Underestimated Key Component of Cell Wall Defence?. Ann. Bot..

[40] Bailey T.L., Elkan C. (1994). Fitting a Mixture Model by Expectation Maximization to Discover Motifs in Biopolymers. Proc. Int. Conf. Intell. Syst. Mol. Biol..

[41] Cortes T., Schubert O.T., Rose G., Arnvig K.B., Comas I., Aebersold R., Young D.B. (2013). Genome-Wide Mapping of Transcriptional Start Sites Defines an Extensive Leaderless Transcriptome in Mycobacterium Tuberculosis. Cell Rep..

[42] Danino Y.M., Even D., Ideses D., Juven-Gershon T. (2015). The Core Promoter: At the Heart of Gene Expression. Biochim. Biophys. Acta.

[43] Zhang H., Yu F., Xie P., Sun S., Qiao X., Tang S., Chen C., Yang S., Mei C., Yang D. (2023). A Gγ Protein Regulates Alkaline Sensitivity in Crops. Science.

[44] Smirnoff N., Arnaud D. (2019). Hydrogen Peroxide Metabolism and Functions in Plants. New Phytol..

[45] Waszczak C., Carmody M., Kangasjärvi J. (2018). Reactive Oxygen Species in Plant Signaling. Annu. Rev. Plant Biol..

[46] Binenbaum J., Weinstain R., Shani E. (2018). Gibberellin Localization and Transport in Plants. Trends Plant Sci..

[47] Wang Z.-P., Xing H.-L., Dong L., Zhang H.-Y., Han C.-Y., Wang X.-C., Chen Q.-J. (2015). Egg Cell-Specific Promoter-Controlled CRISPR/Cas9 Efficiently Generates Homozygous Mutants for Multiple Target Genes in Arabidopsis in a Single Generation. Genome Biol..

[48] Clough S.J., Bent A.F. (1998). Floral Dip: A Simplified Method for Agrobacterium-Mediated Transformation of Arabidopsis Thaliana. Plant J. Cell Mol. Biol..

[49] Hartig S.M. (2013). Basic Image Analysis and Manipulation in ImageJ. Curr. Protoc. Mol. Biol..

[50] Kumar S., Stecher G., Li M., Knyaz C., Tamura K. (2018). MEGA X: Molecular Evolutionary Genetics Analysis across Computing Platforms. Mol. Biol. Evol..

[51] Livak K.J., Schmittgen T.D. (2001). Analysis of Relative Gene Expression Data Using Real-Time Quantitative PCR and the 2(-Delta Delta C(T)) Method. Methods San Diego Calif..

[52] Chen S., Zhou Y., Chen Y., Gu J. (2018). Fastp: An Ultra-Fast All-in-One FASTQ Preprocessor. Bioinforma. Oxf. Engl..

[53] Kim D., Langmead B., Salzberg S.L. (2015). HISAT: A Fast Spliced Aligner with Low Memory Requirements. Nat. Methods.

[54] Pertea M., Pertea G.M., Antonescu C.M., Chang T.-C., Mendell J.T., Salzberg S.L. (2015). StringTie Enables Improved Reconstruction of a Transcriptome from RNA-Seq Reads. Nat. Biotechnol..

[55] Love M.I., Huber W., Anders S. (2014). Moderated Estimation of Fold Change and Dispersion for RNA-Seq Data with DESeq2. Genome Biol..

[56] Wu T., Hu E., Xu S., Chen M., Guo P., Dai Z., Feng T., Zhou L., Tang W., Zhan L. (2021). clusterProfiler 4.0: A Universal Enrichment Tool for Interpreting Omics Data. Innov. Camb. Mass.

