# Supplementary material for: The AP2/ERF Transcription Factor ERF56 Negatively Regulating Nitrate-Dependent Plant Growth in Arabidopsis
Source: Int J Mol Sci. 2025 Jan 13;26(2):613. doi: 10.3390/ijms26020613 (PMC11765960; doi:10.3390/ijms26020613)
Supplement: Supplementary file 1 [file ijms-26-00613-s001.zip › figureS.pdf]

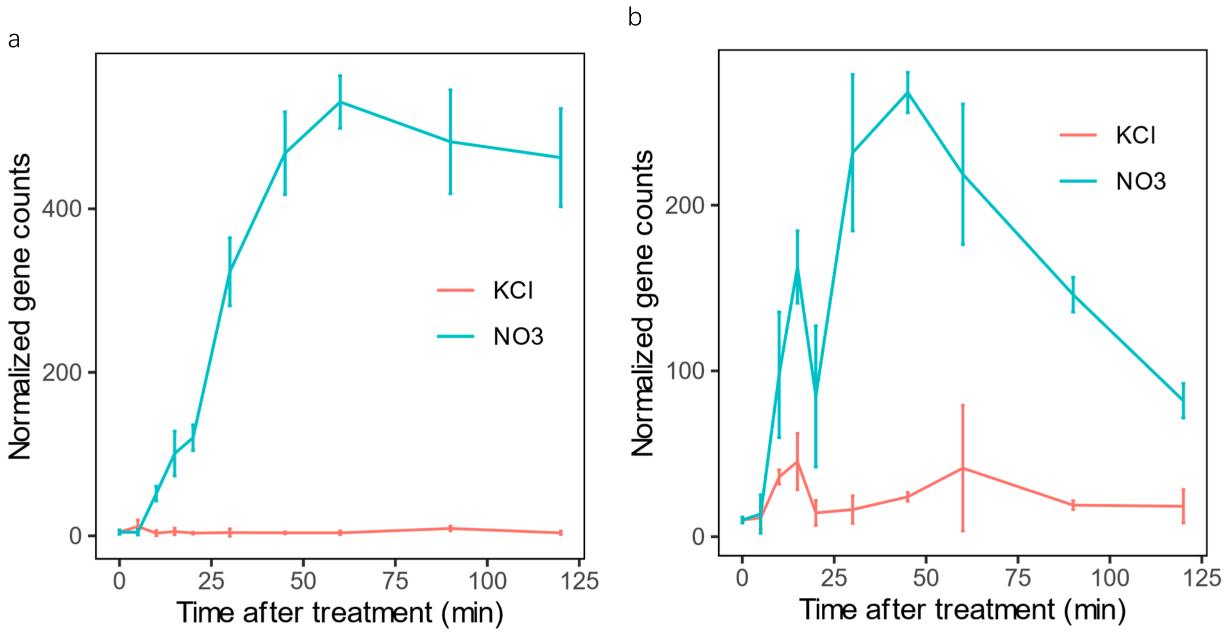

Figure S1. Dynamical mRNA level of *ERF56* in response to NO_3^-. (a) gene counts in shoots of *Arabidopsis*. (b) gene counts in roots of *Arabidopsis*. Data are means ± SD. mRNA level were detected with transcriptome analysis by Varala *et al*. (2018).

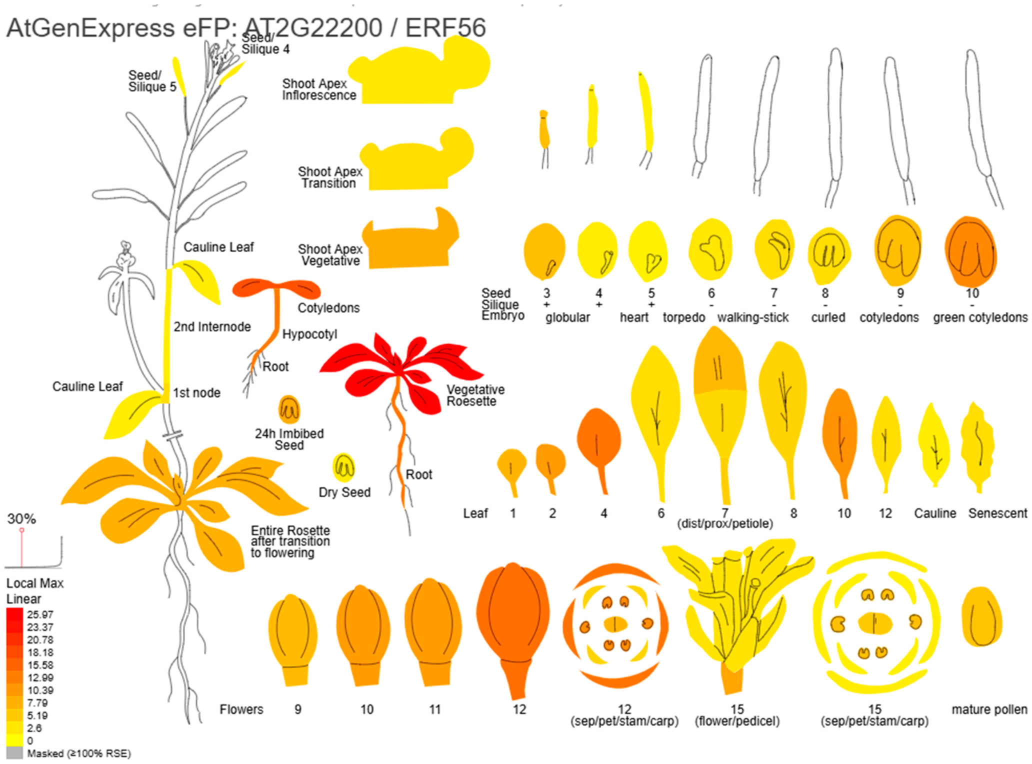

Figure S2. mRNA levels of *ERF56* in different tissues. Data were obtained from https://bar.utoronto.ca/eplant.

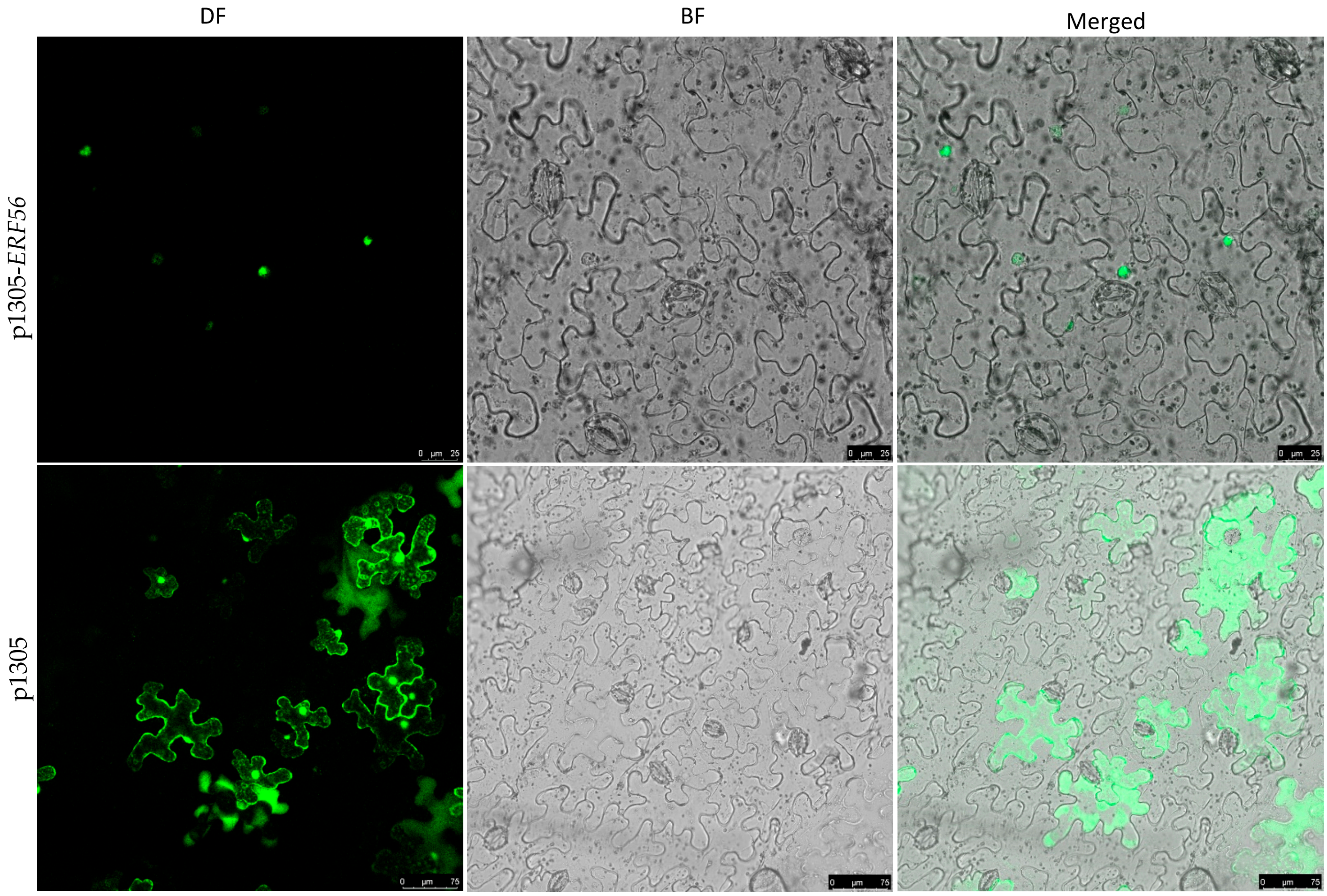

Figure S3. ERF56 is a nucleu-localized protein. Confocal imaging epidermal cells of leaves of *Nicotiana benthamiana* which were infiltrated with *Agrobacteria* containing the plastid carrying *ERF56* (p1305-*ERF56*) or the empty control plastid (p1305). In the plasmid carrying *ERF56*, the CDS of *ERF56* was cloned into the NcoI site of a modified version of the vector pCAMBIA1305 (p1305) with its C-terminal being fused in frame with *GFP* in the vector. DF, the dark field image with laser-aided confocal laser scanning. BF, the bright field image with transmitted light.

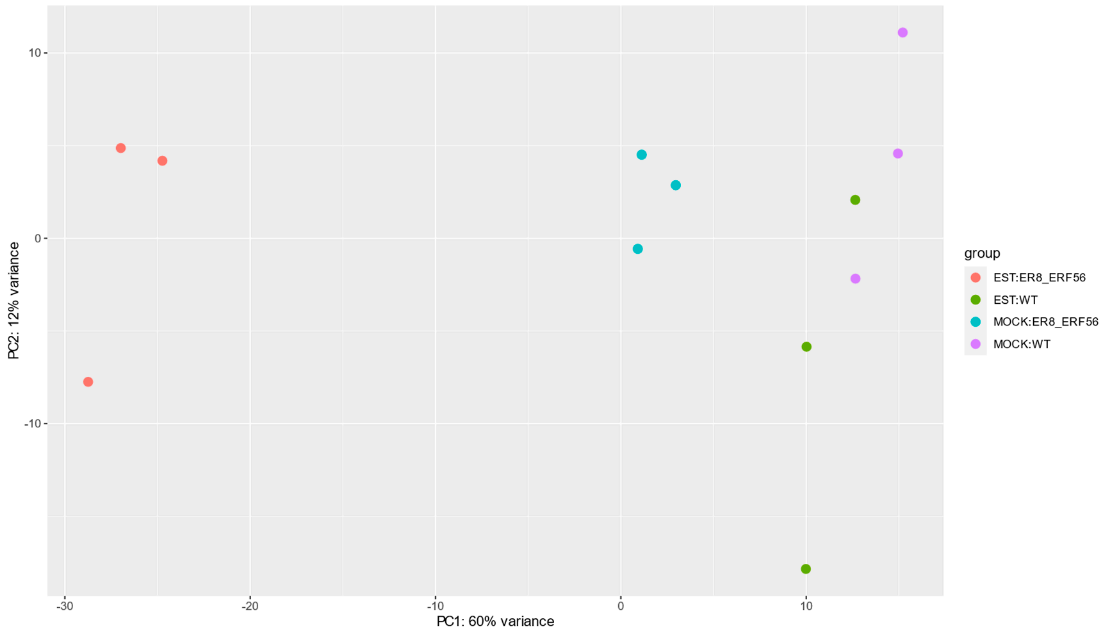

Figure S4. Clustering samples of transcriptome analysis by principal component analysis of gene counts data. For transcriptome assays**,** 10-day-old *ER8-ERF56-1* plants deficient for nitrate were transferred to fresh nitrate-free medium containing 2-uM 17-ß-estradiol (EST) or not, as mock treatment (MOCK), for 3 hours. Each sample had 20 plants. *ER8-ERF56:* an inducible *ERF56* over-expressor. Normalized gene counts with a variance stabilizing transformation in the software DESeq2 were used in the analysis.
